# Acid-modified biochar improves the adaptability of sugar beet seedlings to saline-alkali soil by enhancing their physiology

**DOI:** 10.1186/s12870-026-09769-z

**Published:** 2026-08-20

**Authors:** Chuanhe Zhao, Piao Yunuo, Jean Wan Hong Yong, Muhammad Ishfaq, Muqadas Sattar, Dawei Yin, Muhammad Riaz, Songlin Yang, Baiquan Song

**Affiliations:** 1 Engineering Research Center of Agricultural Microbiology Technology, Ministry of Education & Heilongjiang Provincial Key Laboratory of Ecological Restoration and Resource Utilization for Cold Region & School of Life Sciences, College of Advanced Agriculture and Ecological Environment, National Beet Medium-Term Gene Bank, Heilongjiang University, Harbin, 150080 China; 2 College of Life Sciences, Heilongjiang Bayi Agricultural University, Daqing, 163319 China; 3 Department of Biosystems and Technology, Swedish University of Agricultural Sciences, Alnarp, 23456 Sweden; 4 Faculty of Science and Engineering, Brightlands Future of Farming Institute, Maastricht University, Venlo, 5928 SX The Netherlands; 5 College of Life Sciences and Oceanography, Shenzhen University, Shenzhen, 518060 China; 6 Department of Agricultural Engineering, Khwaja Fareed University of Engineering and Information Technology, Rahim Yar Khan, Punjab 64200 Pakistan; 7 College of Agricultural Science, Heilongjiang Bayi Agricultural University, Daqing, 163319 China; 8 College of Resources and Environment, Huazhong Agriculture University, Wuhan, 430070 China

**Keywords:** Biochar, Acid modification, Chlorophyll fluorescence, Saline-alkali soil, Sugar beet

## Abstract

Biochar is widely recognized for enhancing plant stress tolerance, and using acid modified-biochar may further enhance its suitability as a soil amendment for plant growth under saline–alkali conditions. However, the underlying mechanisms by which modified biochar regulate sugar beet (*Beta vulgaris* L.) growth and physiology remain unclear. Here, a pot experiment was conducted to evaluate the effects of acid-modified biochar on the physiology of sugar beet seedlings grown in saline–alkali soil. The unfavourable saline–alkali conditions decreased photosynthetic efficiency and inhibited leaf development, whereas all acid-modified biochar treatments restored sugar beet physiological performance, with sulfuric acid–modified biochar (BS) delivering the strongest favourable effects. BS increased the total foliar chlorophyll content by two folds, enhanced the net photosynthetic rate by 86.22%, and improved the photosynthetic performance index (PI_ABS_) and maximum photochemical efficiency (F_v_/F_m_) by 1.5 folds, and 4.13%, respectively. BS also increased the chlorophyll fluorescence energy flux parameters (RE_0_/CS_m_, ET_0_/CS_m_, and ET_0_/CS_0_). In addition, BS lowered leaf relative electrical conductivity by 6.79%, decreased superoxide dismutase (SOD) activity by 23.53%, and increased catalase (CAT) activity by 22.43%; suggesting reduced membrane damage and potentially a more balanced antioxidant response. As a result, leaf area and aboveground biomass increased by 15.61% and 139.44%, respectively. Taken together, these findings demonstrated that sulfuric acid–modified biochar amendment mitigated the saline–alkali stress in sugar beet by stabilizing foliar PSII activity, optimizing energy allocation, and maintaining membrane integrity, and providing the physiological basis for restoring sugar beet growth in unfavourable saline–alkali soils.

**Graphical Abstract:**

**Figure:**
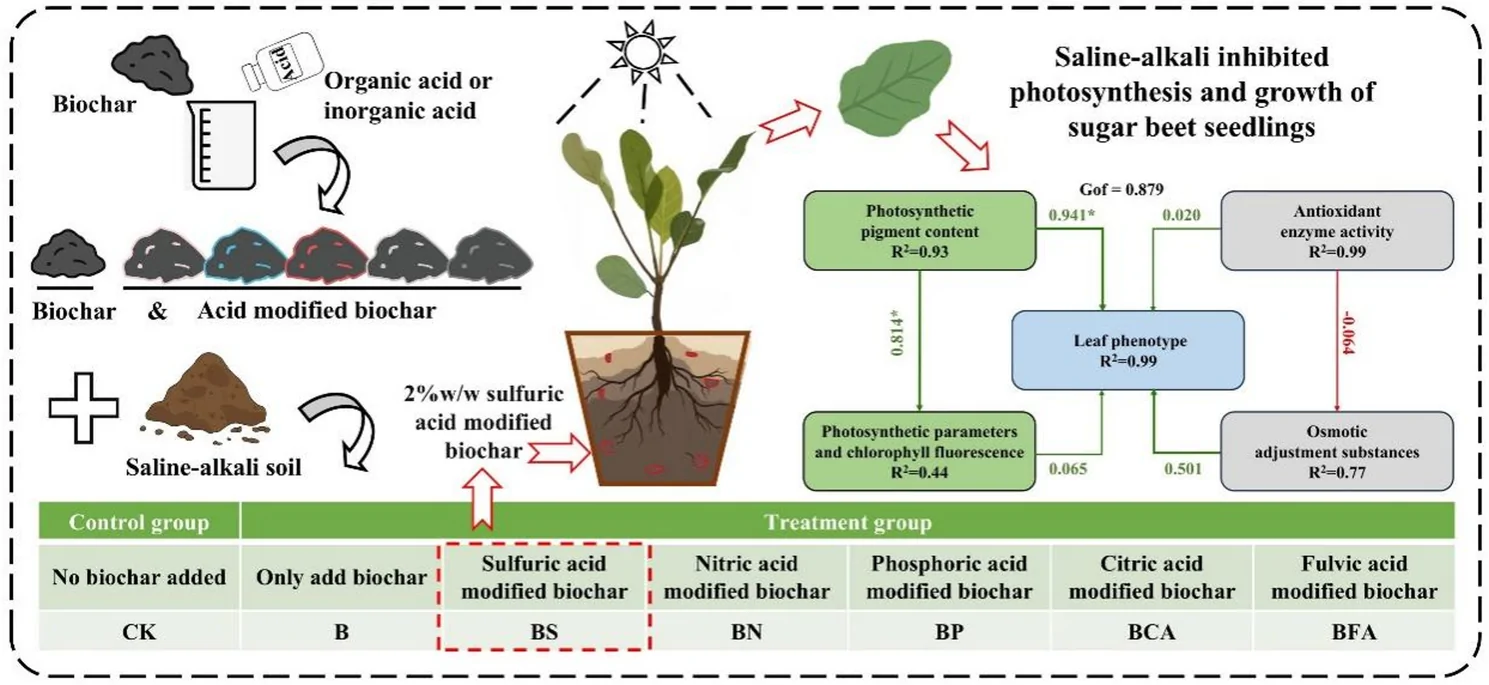

**Supplementary Information:**

The online version contains supplementary material available at 10.1186/s12870-026-09769-z.

## Introduction

Sugar beet (*Beta vulgaris* L.) is a major sugar crop of high economic value, contributing approximately 20% of global sugar production and serving as a key component of the world sugar supply [1]. In China, sugar beet is an important sugar crop in the arid and semi-arid regions of the north. Its annual output has reached several million tons, and the planting area exceeds 200,000 hectares, making it a vital driver of local agricultural income and promoting regional economic stability [2]. The saline–alkali condition in soils is one of the primary environmental constraints limiting high-quality, high-yield sugar beet production. Globally, more than one billion hectares, about 10–11% of the earth’s land surface, are affected by salinity, and this figure continues to expand. China alone has nearly 100 million hectares of saline-alkali land, posing a great challenge to agricultural sustainability. In major sugar beet production zones, soil salinity in roughly 20% of cultivated land already exceeds the crop’s tolerance threshold, severely reducing growth and productivity [3]. Although sugar beet is more tolerant of salinity and alkalinity than many other crops, its seedling stage remains particularly vulnerable. Under 280 mmol L^-1^ NaCl stress, the chlorophyll content of sugar beet seedlings can decline by up to 38.4% [4]. Salt stress also markedly inhibits seedling growth, biomass accumulation, and photosynthetic efficiency [5]. In China’s main sugar beet-producing regions, the unfavourable saline-alkali have lowered yields by 15–20%, and in severely affected areas, losses can reach 30% [6]. Gaining insights into the physiological responses of sugar beet seedlings to this specific abiotic stress factor is therefore important for developing effective management strategies and ensuring stable production in regions afflicted by saline-alkali soils.

Biochar, with its highly porous structure and abundant surface functional groups, is used to mitigate saline–alkali stress and enhance plant physiological performance [7, 8]. Unlike conventional chemical applications, which often offer only short-term benefits and may even contribute to soil compaction, biochar provides more persistent and environmentally sustainable improvements to soil quality [9–11]. Previous research has shown that biochar alleviates the negative effects of salinity and alkalinity by reducing soil electrical conductivity, adsorbing soluble salt ions, and improving the soil microenvironment, thereby supporting crop growth and photosynthetic function [12]. In addition, biochar can reinforce the plant antioxidant defense system, limit the accumulation of ROS, and improve the structural stability and energy conversion efficiency of photosystem II [13]. Nevertheless, biochar effectiveness varies widely across saline–alkali environments, largely due to differences in feedstock properties, pyrolysis temperatures, and the availability of surface-active sites [14]. To address these limitations, chemical modification has gained attention to enhance biochar functionality. Methods such as acid–base treatment, metal or nutrient loading, and oxidative activation can increase the abundance of oxygen-containing functional groups, expand the surface area, and enrich surface active sites [15–17]. These improvements enhance ion-exchange capacity and pollutant adsorption, thereby optimizing the rhizosphere environment and strengthening plant tolerance to saline–alkali conditions [18].

Among chemical modification strategies, acid-modified biochar has received considerable attention for its strong ion-regulating capacity and its effectiveness in alleviating plant stress [19]. Acid treatment can substantially alter the surface chemistry and acidity of biochar by increasing polar functional groups, thereby enhancing its cation-exchange capacity [18]. In various cropping systems, acid-modified biochar improves ion homeostasis, reduces soil electrical conductivity and exchangeable sodium levels, and strengthens cell membrane stability, all of which lead to greater crop resilience under saline–alkali conditions [20]. In addition, modified biochar promotes the accumulation of photosynthetic pigments and mitigates oxidative stress associated with excessive reactive oxygen species (ROS), supporting the structural integrity of photosystem II (PSII) and improving energy conversion [21]. Changes in chlorophyll fluorescence parameters, chlorophyll content, and gas-exchange characteristics are widely used as indicators of photosynthetic performance and energy allocation in plants under saline–alkali soil [22]. Because sugar beet seedlings are particularly sensitive to environmental stress, their photosynthetic capacity is closely tied to subsequent growth and sugar-accumulation potential. Therefore, elucidating how acid-modified biochar influences the photosynthetic physiology of sugar beet is critical for understanding its role in mitigating saline–alkali stress.

Therefore, this study employed several acid-modification methods to produce different biochar treatments and comparatively assess their effects on sugar beet seedlings. Based on previous findings, we hypothesized that incorporating acid-modified biochar would mitigate saline–alkali stress, improve physiological performance, and ultimately restoring growth under unfavourable conditions. Accordingly, this study aimed to clarify how various types of modified biochar influence the growth and physiology of sugar beet under saline–alkali conditions, while also examining changes in antioxidant activity and membrane permeability. The results are expected to support the targeted use of soil amendments in sugar beet production in regions with saline–alkali soils and offering mechanistic insights into the role of biochar in soil rehabilitation.

## Materials and methods

### Experiment site and material preparation

The experiment was conducted in an artificial-light growth chamber at the National Sugar Crop Improvement Center (Harbin, China). The sugar beet variety used was KWS1176, supplied by KWS SAAT SE & Co. KGaA (Germany), and currently one of the major cultivars grown in Northeast China. Soil was collected from saline–alkali fields in Zhaoyuan County, Daqing City, Heilongjiang Province (45°23′N, 123°47′E) from a depth of 5–20 cm. The soil of the top 5 cm was removed, and the remaining portion was homogenized, air-dried, sieved (2 mm), and stored for subsequent analysis.

Biochar was produced from corn stover pyrolyzed in a muffle furnace (SX-4-10, Tianjin, China) at 500℃ with a heating rate of 10℃ min^-1^ and a retention time of 2 h. After pyrolysis, the biochar was air-dried, ground, and sieved to 0.85 mm. The resulting material had a pH of 7.86 and an organic carbon content of 45.8%. For acid modification, both inorganic and organic acid solutions were prepared. Phosphoric, sulfuric, and nitric acids were diluted with distilled water to a final H^+^ concentration of 1 mol L^-1^, while fulvic and citric acids were dissolved and adjusted to the same concentration. For each modification treatment, 900 g of corn stover biochar was added to 9 L of the respective acid solution (solid-to-liquid ratio of 1:10, w/v) in a 15 L glass container. The mixture was continuously stirred at 200 rpm using an overhead mechanical stirrer at room temperature (25 ± 2℃) for 24 h, with additional manual stirring every 2 h to prevent settling. After soaking, the biochar was separated by vacuum filtration and rinsed repeatedly with distilled water until the pH of the filtrate reached 6.5–7.0. The acid-treated biochar was then air-dried at room temperature and stored in sealed bags until use [23–26].

### Experimental design and methods

Seven treatments were established under controlled potting conditions: untreated saline–alkali soil (CK); saline–alkali soil amended with 2% biochar (B); 2% phosphoric acid–modified biochar (BP); 2% sulfuric acid–modified biochar (BS); 2% nitric acid–modified biochar (BN); 2% fulvic acid–modified biochar (BFA); and 2% citric acid–modified biochar (BCA). The biochar was applied based on earlier studies and meta-analyses conducted in similar soil environments [27–30]. Each treatment consisted of 10 replicates.

Before sowing, each type of biochar was thoroughly mixed into the saline–alkali soil (Li et al., 2022). The experiment was conducted in pots containing 500 g of soil (diameter: 8 cm, height: 10 cm), with one sugar beet seedling planted per pot. All pots were placed in a greenhouse under natural light, with an intensity of 7000 lx, day/night temperatures of 26 °C/20°C, a 12 h photoperiod, relative humidity of 45%, and a soil relative water content was maintained at 30%.

### Sampling methods and measurement items

#### Determination of plant morphological parameters

Forty-five days after emergence, uniformly growing sugar beet seedlings were harvested. Plant height was measured using a ruler, and fully expanded compound leaves were collected to determine leaf width and length. Leaf area (LA) and specific leaf area (SLA) were then calculated accordingly [31].

1 $$\mathrm{LA}=\mathrm W\times\mathrm L\times0.754$$

where W and L represent leaf width and length, respectively.

2 $$\mathrm{SLA}=\mathrm{LA}/\mathrm M$$

where M denotes leaf dry mass.

#### Photosynthetic pigments, fluorescence parameters, and gas exchange parameters

Homogenized fresh leaf samples (0.1 g) were extracted with 10 mL of 95% ethanol in darkness at 4 °C for 24 h. The extracts absorbance was measured at 665, 649, and 470 nm using a UV spectrophotometer (UV-8000 A, Shanghai, China), and chlorophyll concentrations were determined.

For chlorophyll fluorescence measurements, the middle section of each leaf was dark-adapted for 15 min using a dark adaptation clip. OJIP transients and light–dark adaptation parameters were recorded using a Hansatech PocketPEA high-speed fluorometer, with three biological replicates per treatment. Photosystem II (PSII) performance, including the photosynthetic performance index (PI_ABS_) and maximum photochemical efficiency (F_v_/F_m_) was analyzed based on the OJIP test [32].

Fully expanded compound leaves were selected, and gas exchange parameters were recorded from at least three plant leaves per treatment with comparable growth vigor, similar leaf positions, and consistent orientation. Measurements were conducted using a portable photosynthesis system (model TARGAS-1, PP Systems, Amesbury, USA) at 9:00 and 11:00 a.m. The measured parameters included transpiration rate (E), net photosynthetic rate (Pn), intercellular CO₂ concentration (Ci), and stomatal conductance (Gs) [33].

#### Plant stress physiological indicators

The hydrogen peroxide (H_2_O_2_) and superoxide anion (O_2_^-^) accumulation in leaves were determined using 3,3′-diaminobenzidine (DAB) staining and nitroblue tetrazolium (NBT), respectively. Leaves from the same position in each treatment were selected, soaked, stained, and subsequently rinsed and decolorized with ethanol.

For leaf antioxidant enzyme activities determination, superoxide dismutase (SOD) activity was measured using the NBT reduction [34], peroxidase (POD) activity was assessed using the guaiacol [35], and catalase (CAT) activity was quantified with the ultraviolet absorption method [36]. The thiobarbituric acid method was used to determine malondialdehyde (MDA) content [37]. In addition, osmotic adjustment substances were analyzed, and the anthrone colorimetric method was used to measure soluble sugar content [38].

### Data statistics and analysis

All collected data were organized and preliminarily processed using Microsoft Excel 2020 (Microsoft Corporation, Redmond, WA, USA), and statistical analyses were subsequently performed with IBM SPSS Statistics 25 (IBM Corporation, Armonk, NY, USA). Graphical visualization and figure preparation were carried out using OriginPro 2021 (OriginLab Corporation, Northampton, MA, USA) in combination with Microsoft PowerPoint (Microsoft Corporation, Redmond, WA, USA). A one-way analysis of variance (ANOVA) was conducted to assess the statistical significance among treatments, followed by Tukey’s multiple range test for post hoc pairwise comparisons. Data represent means ± SD of five biological replicates per treatment. Statistical differences (*P* < 0.05) among means are denoted by different alphabetical labels (a, b, c). Furthermore, to explore structural associations among physiological indicators and photosynthetic parameters, partial least squares path modeling (PLS-PM) was performed using the *plspm* package in R software (version 4.5.1).

## Results and analysis

### Effects of different treatments on the leaf phenotype of sugar beet seedlings

The acid-modified biochar effectively promoted sugar beet growth under saline-alkali stress and markedly enhanced aboveground biomass. Among all treatments, sulfuric acid-modified biochar (BS) exhibited the strongest effect. Compared with the CK, leaves under biochar treatments were significantly wider and exhibited greater developmental vigor.

**Fig. 1:**
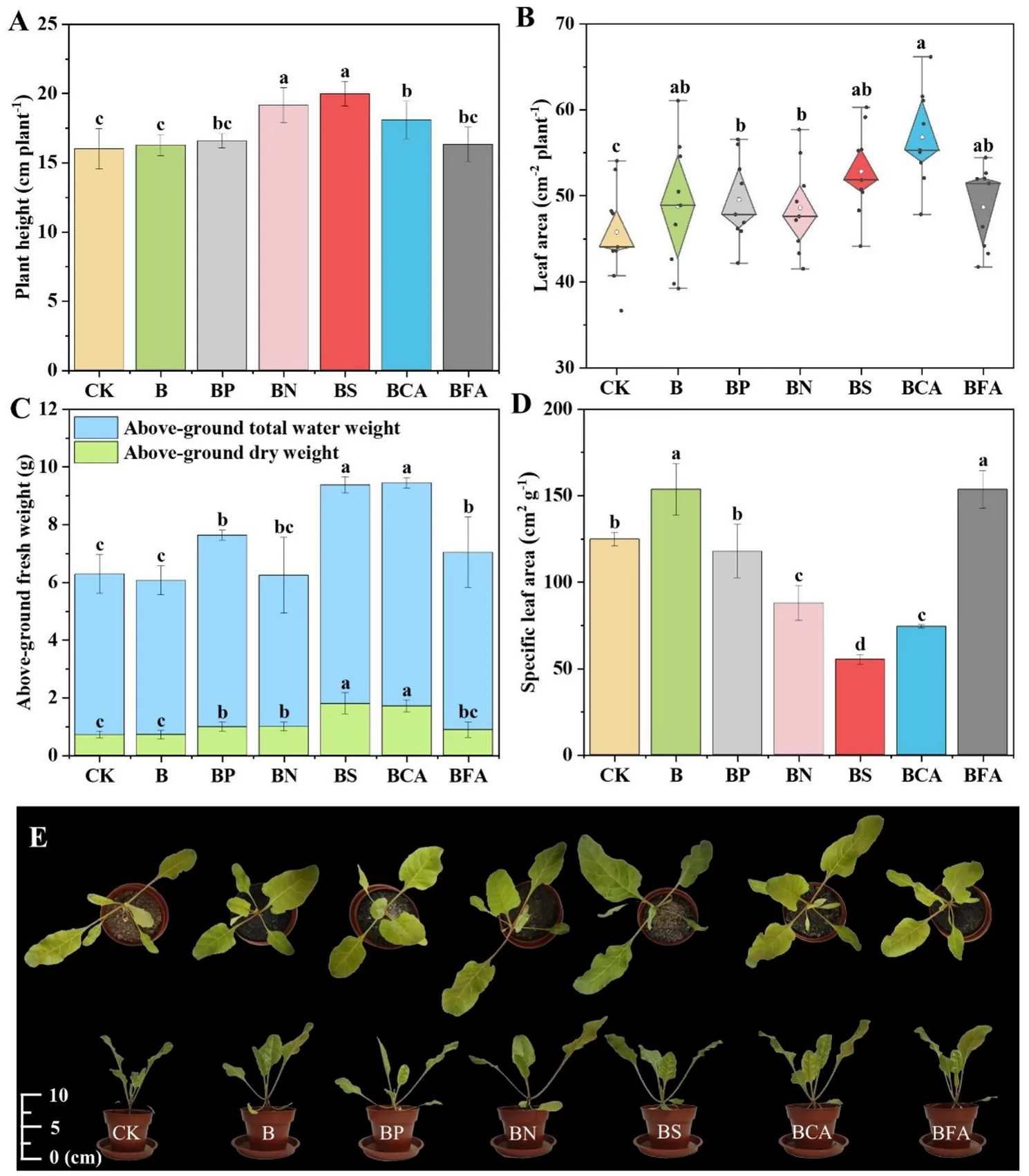
Effects on sugar beet seedlings. **A **Plant height; (**B**) Leaf area; (**C**) Aboveground dry and fresh weights; (**D**) Specific leaf area; (**E**) Top and front views of seedlings under different treatments

Relative to CK, the BS treatment increased plant height and leaf area by 24.75% and 15.61%, respectively. The citric acid-modified biochar (BCA) treatment showed a similar trend, with plant height and leaf area increasing by 12.94% and 25.55%, respectively (Fig. 1A-B). Furthermore, the BS treatment produced the greatest increases in aboveground dry weight and fresh weight, reaching 139.44% and 34.30%, respectively (Fig. 1C). Although biochar addition generally increased specific leaf area, all acid-modified biochar treatments resulted in a decrease in SLA, with the BS treatment exhibiting the most pronounced reduction of 55.75% (Fig. 1D).

### Effects on photosynthetic pigment content and photosynthetic parameters

**Fig. 2:**
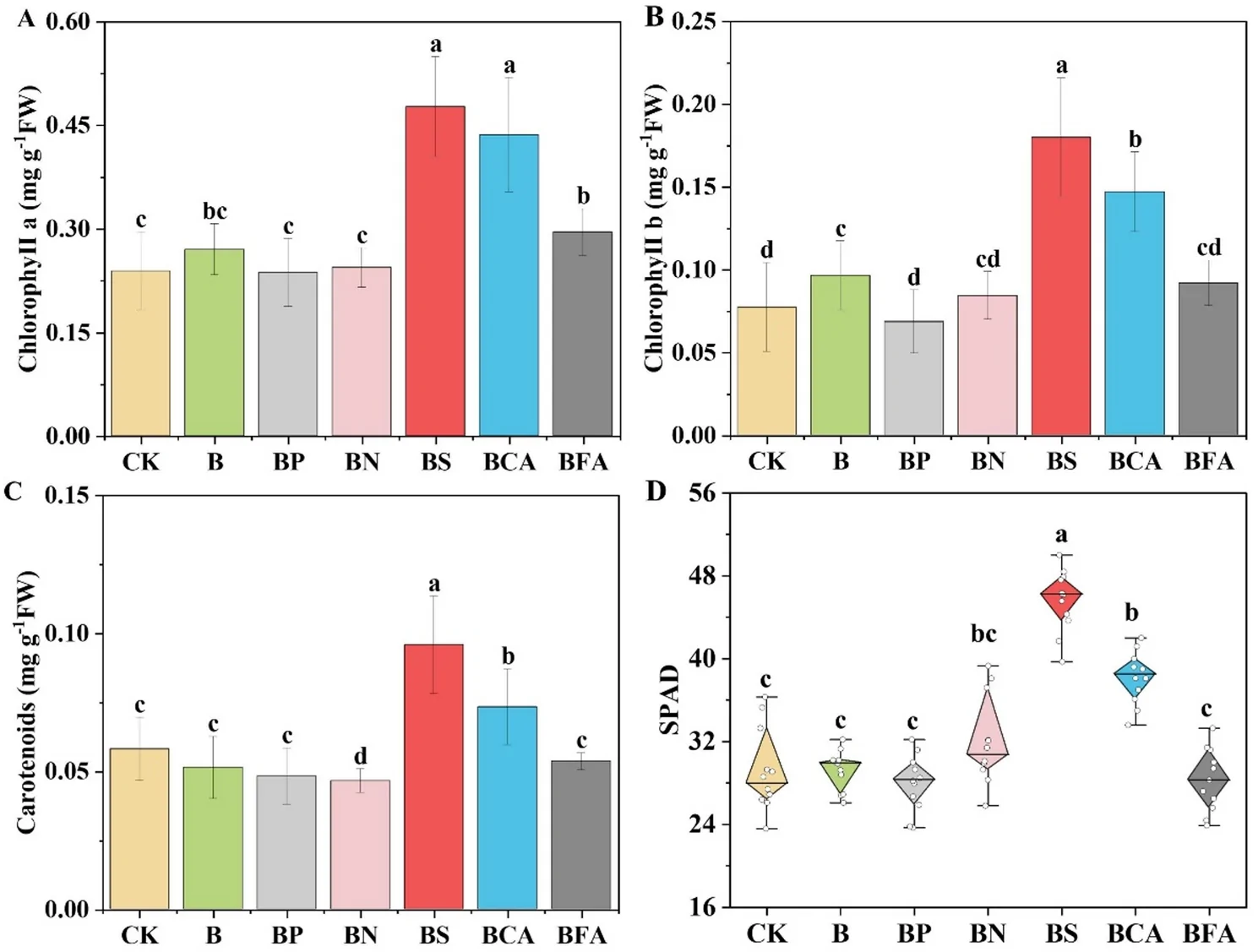
**A**-**C **Chlorophyll a, chlorophyll b, and carotenoid contents of sugar beet leaves; (**D**) Leaf SPAD values

Compared with CK, the application of unmodified biochar (B) only significantly increased chlorophyll b content by 24.74%, with little change observed in the other pigments. In contrast, specific acid-modified biochars markedly enhanced chlorophyll content. Notably, after the BS application, chlorophyll a, chlorophyll b, and carotenoid contents increased by 99.63%, 132.61%, and 64.95%, respectively, resulting in a total chlorophyll increase of 205.07% compared with CK. Similarly, BCA treatment significantly elevated the three pigments by 82.65%, 90.13%, and 26.25%, respectively, whereas other modified biochars did not show consistent effects (Fig. 2, A-C). The SPAD values exhibited a trend consistent with chlorophyll content, with both the mean and median SPAD values in the BS treatment being higher than those in all other treatments (Fig. 2, D).

**Fig. 3:**
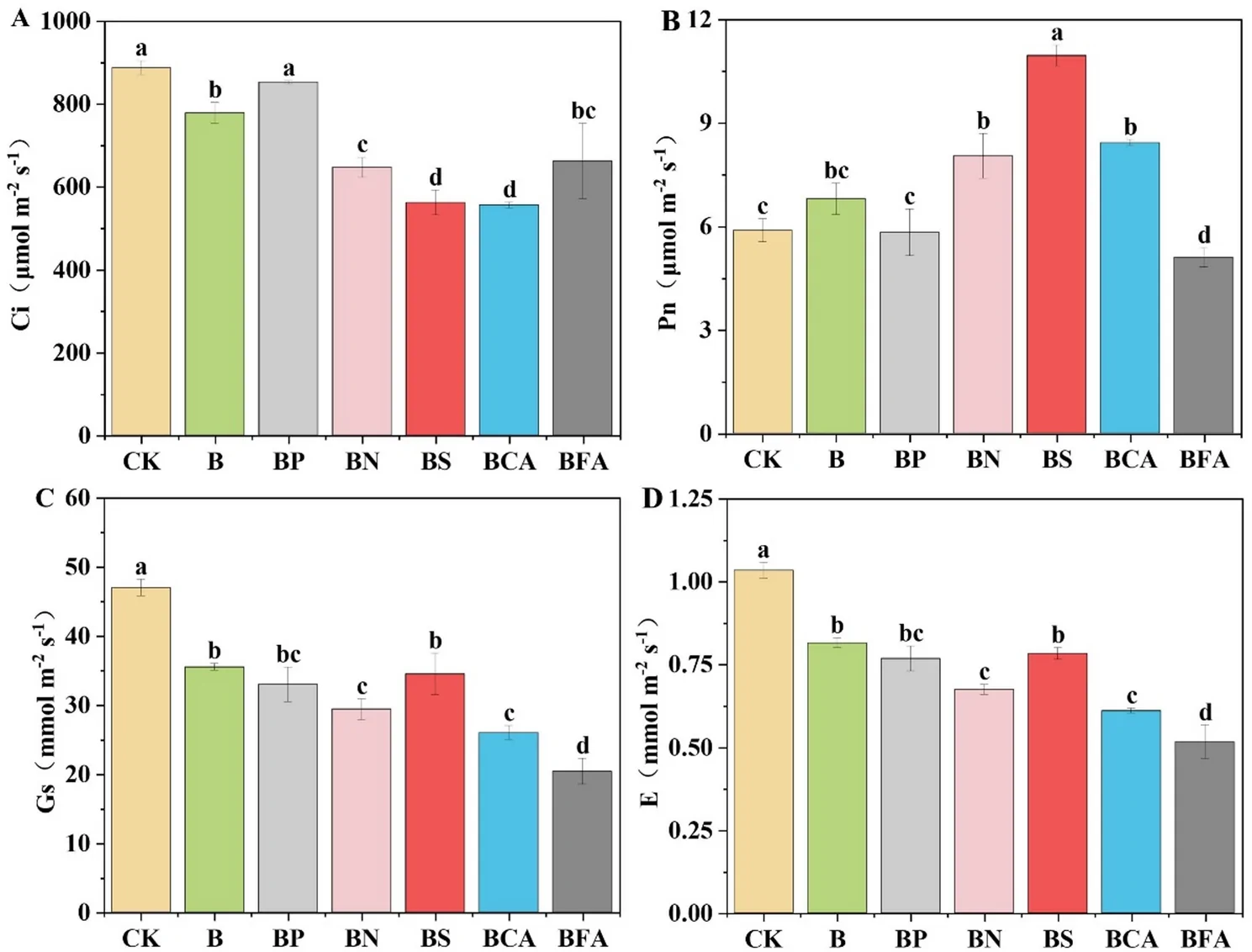
**A**-**D **The gas exchange characteristics of sugar beet seedlings, including net photosynthetic rate (Pn), intercellular CO_2_ concentration (Ci), stomatal conductance (Gs), and transpiration rate (**E**)

Compared with CK, the addition of unmodified biochar (Treatment B) led to significant photosynthetic activity variations except for the net photosynthetic rate (Pn). Sulfuric acid-modified biochar (BS) had an even more pronounced effect, with Pn increasing by 86.22% to CK (*P* < 0.05). In contrast, intercellular CO_2_ concentration (Ci), the transpiration rate (E), and stomatal conductance (Gs) generally decreased across all biochar treatments (Fig. 3A-D). The present results imply that acid modification tends to strengthen the beneficial roles of biochar in maintaining photosynthetic capacity of sugar beet seedlings under saline-alkali stress.

### Effects on foliar chlorophyll fluorescence

**Fig. 4:**
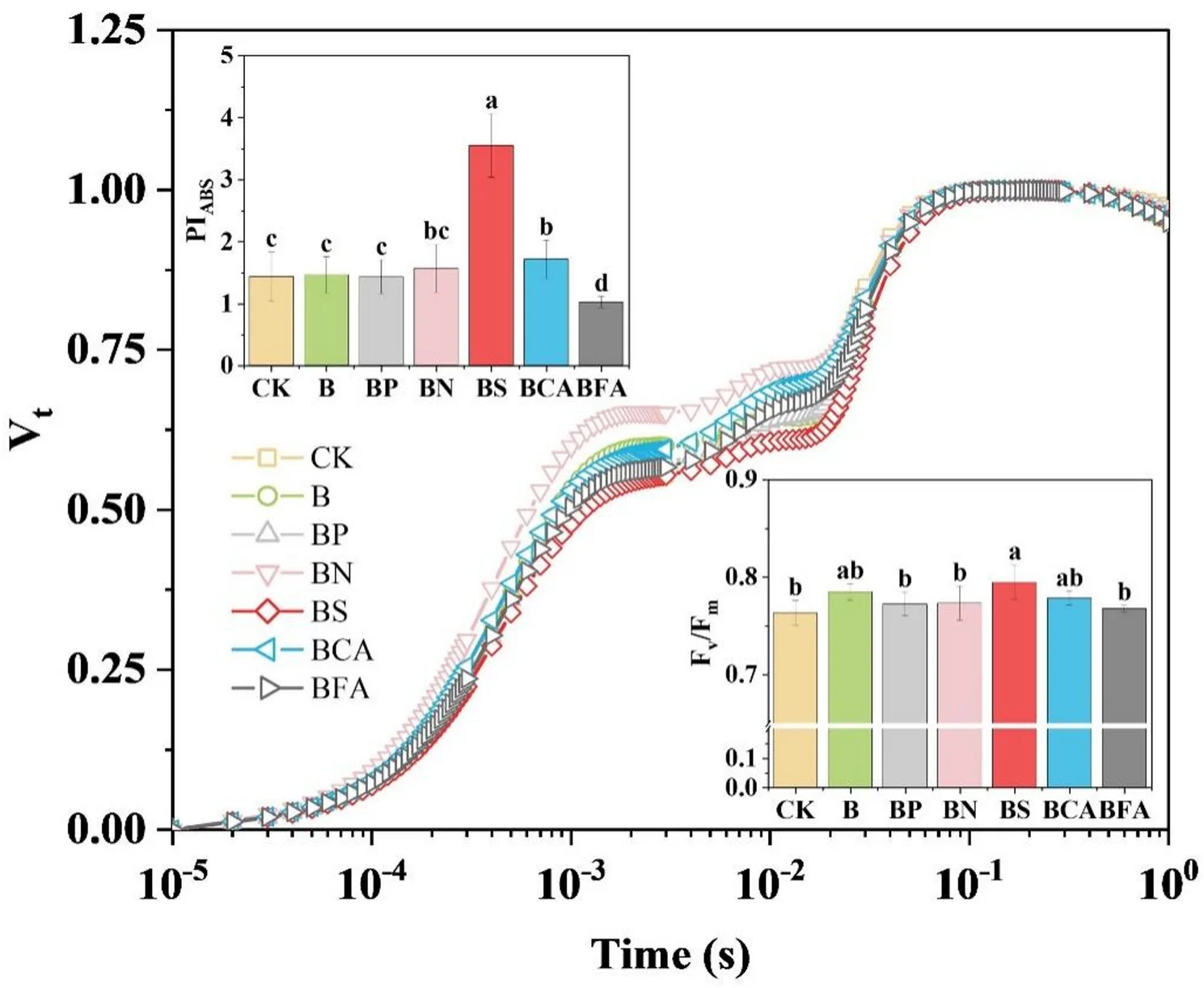
Normalized OJIP fluorescence curves for different treatments and their effects on photochemical activity

The standardized OJIP fluorescence kinetic curves were analyzed for each treatment. The saline-alkali environment (CK) markedly reduced the maximum dark-adapted fluorescence (F_m_), whereas F_m_ increased significantly in most biochar treatments, with sulfuric acid-modified biochar (BS) showing the greatest enhancement. Although the initial fluorescence (F_0_) was slightly higher in all treatments when compared to CK, the differences were not statistically significant (Fig. 4A). Regarding the photosynthetic performance index (PI_ABS_), the BS treatment increased it by 147.35% relative to CK. Additionally, BS enhanced the maximum quantum efficiency of PSII (F_v_/F_m_) by 4.13% (Fig. 4B-C), indicating an incremental elevation in PSII activity under saline-alkali stress.

**Fig. 5:**
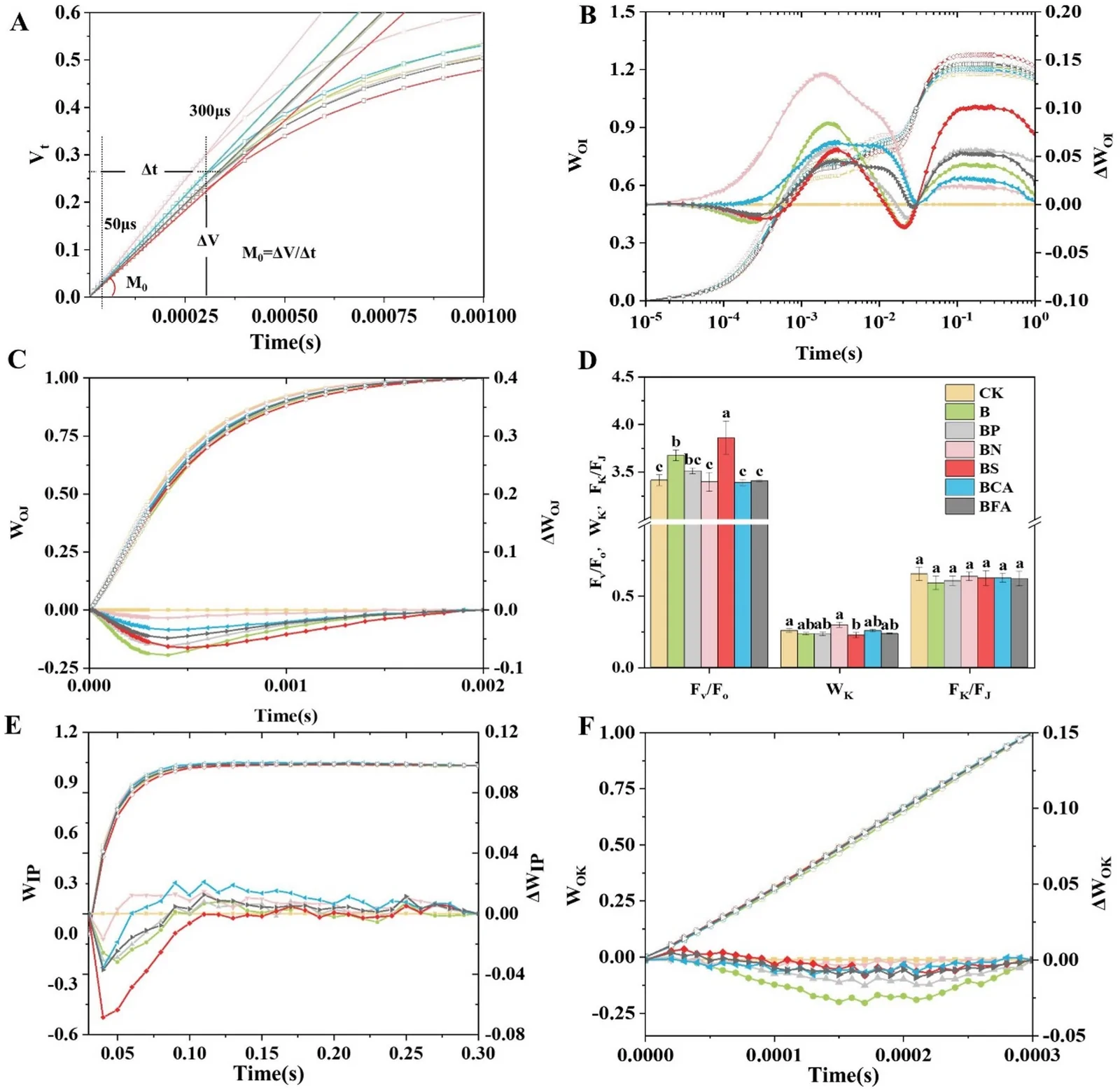
OJIP curves of sugar beet seedlings. **A **Normalized OJIP curves and average initial slope (M0) of relative variable fluorescence of chlorophyll a. **B**, **C **Variation patterns of each treatment in different OJIP segments. **D **Oxygen-evolving complex (OEC) activity in PSII. **E**, **F **Variation patterns of each treatment in the IP and OK segments

Analysis of the early segment of the normalized OJIP curves showed that the average initial slope (M_0_) of relative variable fluorescence was similar, with no significant differences across all treatments (Fig. 5A). Segmental normalization indicated that WOI reflects the relative capacity of the terminal electron acceptor pool on the PSI acceptor side. In this study, WOI values for all modified biochar treatments were below 1, with no significant differences among treatments (Fig. 5B). No pronounced K-band was observed in the OJ phase (TK = 0.0003 s), suggesting that the oxygen-evolving complex (OEC) remained relatively stable under modified biochar application (Fig. 5C). Comparison of OEC activity and electron transfer efficiency (F_v_/F_0_) showed that F_v_/F_0_ in the BS was significantly higher compared to CK, whereas OEC activity and K-band fluorescence intensity did not differ significantly among treatments (Fig. 5D). Similarly, no G-band was detected in the IP phase across treatments (Fig. 5E), and no L-band was observed in the OK phase (TL = 0.00015 s), indicating minimal impact on electron transfer on the PSII acceptor side (Fig. 5F). Overall, the BS treatment maintained stability more effectively than other treatments of the PSII photochemical reaction center and ensured smooth electron transport, consistent with its positive effects on photosynthetic performance and chlorophyll content.

The radar chart of chlorophyll fluorescence parameters showed that the BS treatment outperformed the control in both energy flux per cross-section and specific flux, with particularly notable advantages in light absorption and energy conversion efficiency. Energy flux density indicators, including the electron flow rate per leaf area at maximum fluorescence (RE_0_/CS_m_), electron transport rate at maximum fluorescence (ET_0_/CS_m_), initial electron flow rate per leaf area (RE_0_/CS_0_), and initial electron transport rate per leaf area (ET_0_/CS_0_)—all increased under BS. These results indicate that coordination between photosystem II (PSII) and photosystem I (PSI) was strengthened, the electron transport chain operated more efficiently, and the potential for photosynthetic carbon assimilation was enhanced.

**Fig. 6:**
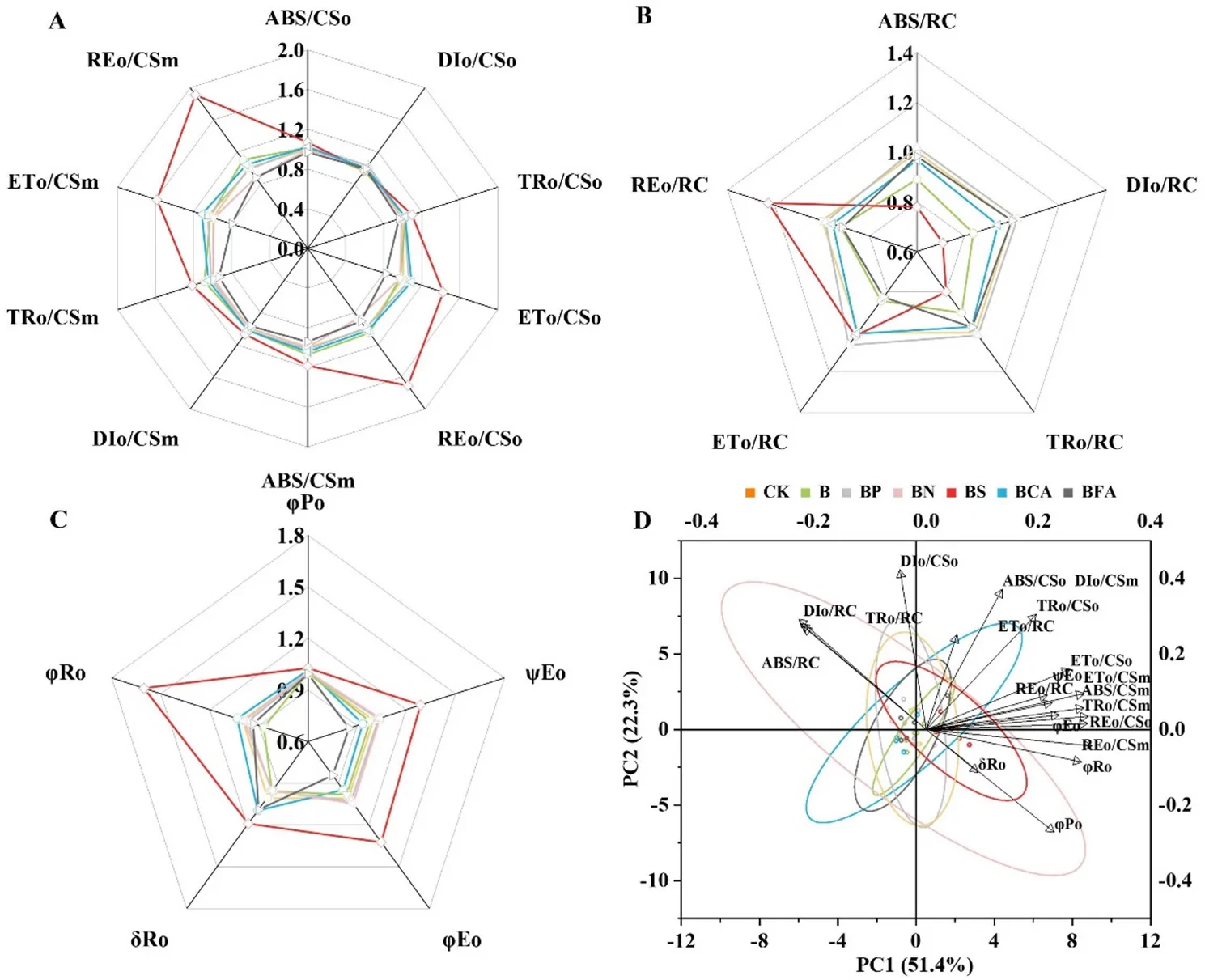
Leaf fluorescence parameter profiles under different treatments. **A **Radar chart of energy flux density indicators. **B **Radar chart of specific energy flux parameters. **C **Radar chart of quantum yield and flux ratio parameters. **D **PCA of JIP-test parameters for each treatment

Further analysis of quantum yield and flux ratios confirmed that the BS treatment improved the light energy utilization efficiency of PSII while reducing energy dissipation caused by non-photochemical quenching (Fig. 6A-C). Principal component analysis (PCA) showed clear differentiation in fluorescence parameter responses among treatments, with BS strongly associated with the quantum efficiency of terminal electron acceptor reduction on the PSI acceptor side (φRo) and the electron flux transferred from a single active reaction center to PSI terminal acceptors (RE_0_/RC) (Fig. 6D).

### Effects of different treatments on antioxidant enzyme activities and osmotic adjustment substances in sugar beet leaves

**Fig. 7:**
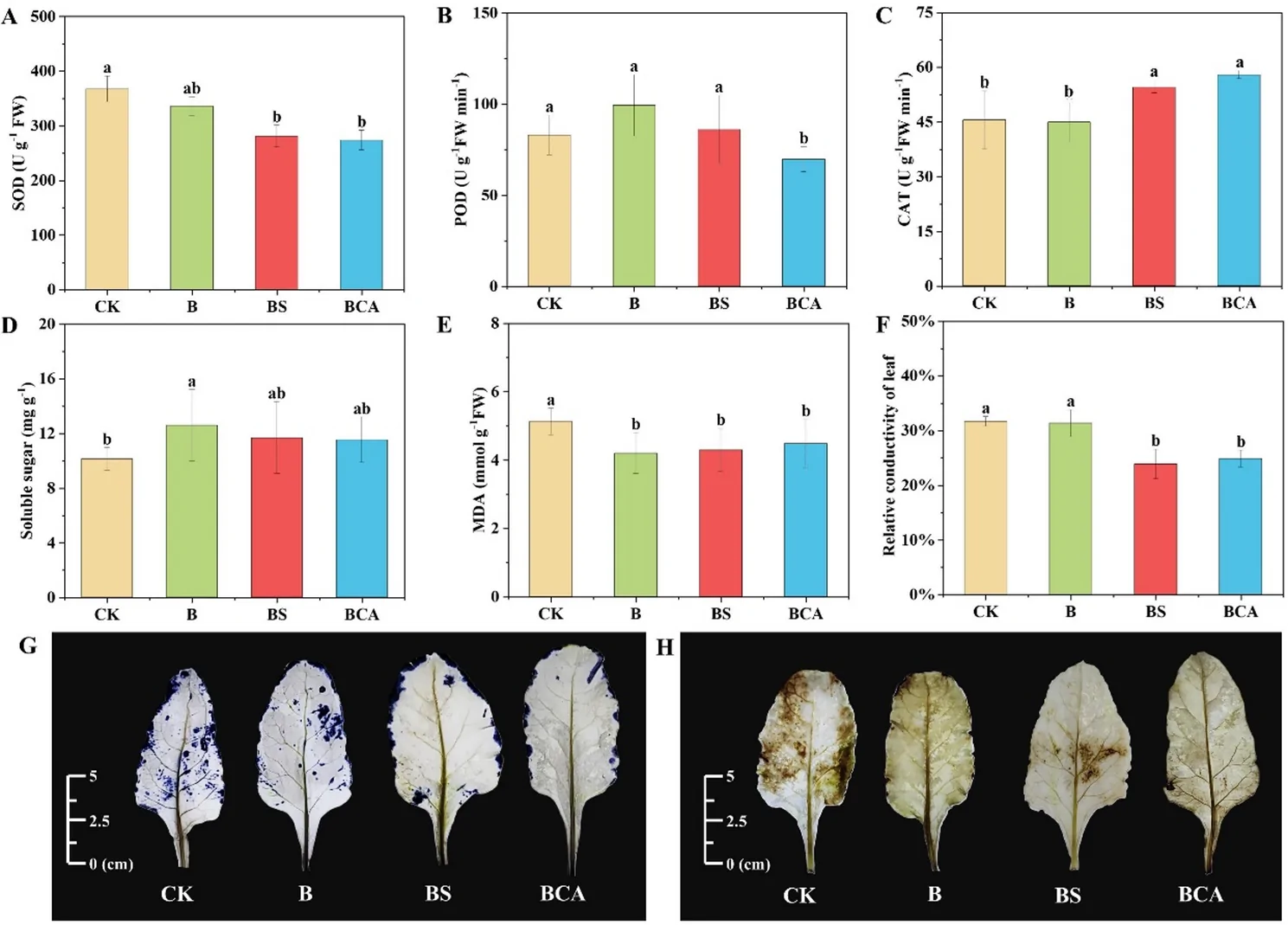
Antioxidant enzyme activities and osmotic adjustment substances in sugar beet leaves. **A**-**C **Activities of SOD, POD, and CAT; (**D**) Soluble sugar content; (**E**) Leaf MDA content; (**F**) Relative electrical conductivity of leaves; (**G**) NBT staining of leaf tissues; (**H**) DAB staining of leaf tissues. As no significant or consistent differences were observed among the acid-modified biochar treatments, only the BS and BCA treatments are presented for comparative visualization alongside biomass and photosynthetic parameters

Compared with the CK, the B treatment caused no significant effect on antioxidant enzyme activities in sugar beet leaves. Conversely, the BS treatment induced a distinct alteration in the antioxidant response, resulting in a 23.53% decrease in SOD activity and a 22.43% increase in catalase (CAT) (Fig. 7A-C). Histochemical staining further supported these findings: nitroblue tetrazolium (NBT) and 3,3′-diaminobenzidine (DAB) staining revealed comparable peroxide (brown) and superoxide anion (blue) accumulation in the CK and B treatments, whereas all acid-modified biochar treatments showed visibly reduced reactive oxygen species (ROS) deposition in leaves (Fig. 7G-H).

Compared with CK, only the unmodified biochar (B) treatment showed significantly higher soluble sugar content in sugar beet leaves, with an increase of 24.41%. The application of acid-modified biochars caused no statistically significant changes in osmotic adjustment substances. Although soluble sugar content under the BS treatment increased by 15.39%, the difference was not significant (Fig. 7D). In addition, biochar addition generally reduced MDA content in leaves, but the decrease was not significant across treatments. Regardless of modification type, the extent of MDA reduction was similar; in the BS treatment, MDA content decreased by 18.01% relative to CK. Notably, the relative EC of leaves in the BS treatment decreased significantly by 6.79% compared with CK, indicating enhanced cell membrane stability under sulfuric acid-modified biochar application (Fig. 7E-F).

### PLS-PM analysis of key physiological indicators under sulfuric acid-modified biochar treatment

**Fig. 8:**
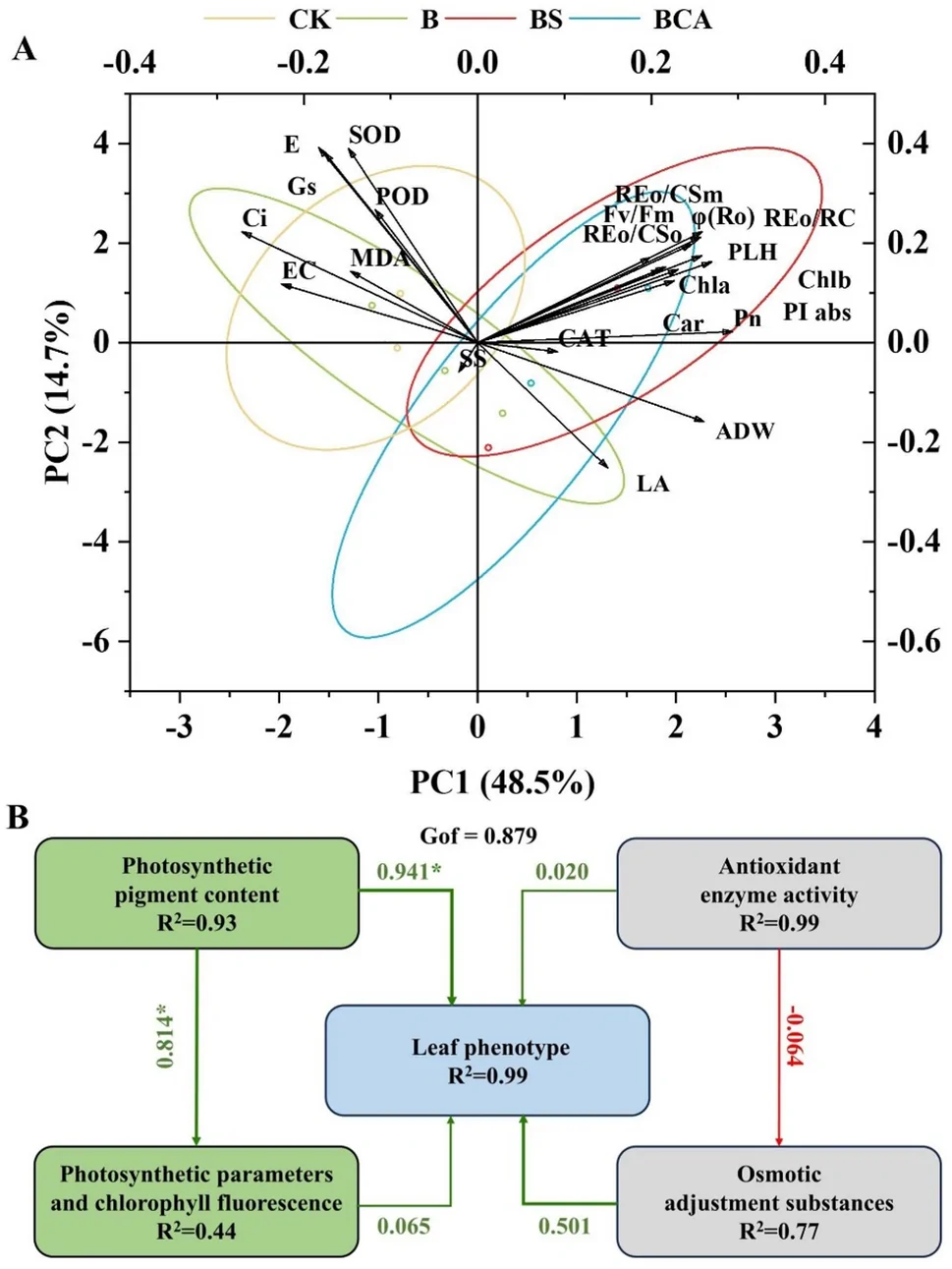
**A **Redundancy analysis (RDA) of major physiological indicators under representative treatments. **B **PLS-PM of key physiological indicators in the sulfuric acid-modified biochar (BS) treatment. Observed variables were grouped into five latent variables, and only those with higher weight contributions are shown. Paths marked with “*” indicate significant correlations. Red and green arrows represent negative and positive correlations, respectively. The thickness of the arrow denotes influence strength, and values indicate standardized path coefficients. Gof represents model goodness of fit

The PLS-PM analysis revealed distinct clustering patterns among treatments. This indicates that sulfuric acid-modified biochar markedly enhanced photosynthetic pigment accumulation, photosynthetic efficiency, and overall plant growth performance under saline-alkali stress (Fig. 8A). In contrast, the CK and unmodified biochar (B) treatments were positioned toward the negative direction of PC1, clustering near variables associated with stress-defense responses. This suggests that, under these treatments, sugar beet seedlings remained strongly affected by saline-alkali stress and relied mainly on antioxidant enzyme activity and stomatal regulation to mitigate its adverse effects.

Several of the aforementioned physiological indicators were further examined using PLS-PM. The PLS-PM results revealed the relationships between the photochemical and antioxidant systems and the morphological performance of sugar beet treated with sulfuric acid-modified biochar under saline-alkali conditions. The analysis indicated that photosynthetic pigment content and osmotic adjustment substances were the primary factors influencing aboveground growth. Moreover, variations in chlorophyll content effectively reflected the structural and functional status of the light-harvesting system. Increases in chlorophyll content enhanced the efficiency of light energy absorption, thereby promoting higher photosynthetic rates and improved chlorophyll fluorescence characteristics (Fig. 8B).

## Discussion

In this study, the effects of modified biochars were systematically evaluated through different chemical methods on the early photosynthetic characteristics of sugar beet seedlings and associated physiological responses under saline–alkali stress. Compared with unmodified biochar, most modified biochars, particularly sulfuric acid–modified biochar (BS), significantly increased Pn, chlorophyll content, and photosynthetic performance index (PI_ABS_). At the same time, modified biochars reduced leaf relative electrical conductivity, indicating improved membrane stability. These observations suggested that modified biochar not only enhances the antioxidant defense capacity of sugar beet under unfavourable saline–alkali conditions but also maintaining foliar photosystem II (PSII) functionality through multiple physiological pathways, ultimately improving the carbon assimilation efficiency of the photosynthetic apparatus.

Saline–alkali stress typically inhibits photosynthesis in sugar beet through two main processes. First, osmotic stress restricts stomatal opening, limiting CO_2_ diffusion into leaves. Second, ion toxicity and excessive ROS damage the chloroplast ultrastructure and disrupting the photosynthetic electron transport chain [39–41]. In the present study, the BS treatment resulted in significant increases in F_v_/F_m_ and PI_ABS_, which reflected an improved capacity to maintain maximum photochemical efficiency and overall PSII performance under saline–alkali stress. All treatments exhibited W_OI_ values below 1, indicating that the terminal electron acceptor pool on the PSI acceptor side remained stable after biochar application. This suggests that the regulatory effects of modified biochar were concentrated within PSII and its upstream electron transport steps, rather than directly affecting PSI stability [42, 43]. Thus, sulfuric acid modification appears to enhance PSII electron transport efficiency by improving photochemical stability and reducing oxidative damage. These improvements promote more effective coordination between PSII and PSI, resulting in higher carbon assimilation and better photosynthetic adaptation under saline–alkali conditions.

Further analysis of the OJIP curves showed that BS improved electron transfer efficiency during the OJ phase compared with the CK, while no L- or K-bands were observed. This indicates minimal structural impairment to the oxygen-evolving complex (OEC) on the PSII donor side and suggests that PSII integrity was well maintained under stress. This preservation of PSII structure and function contributed directly to the observed increases in net photosynthetic rate. These observations align with previous reports that salt stress commonly reduces PSII efficiency and induces abnormal OJIP transients in various crops [44–46]. The findings also reinforce the value of chlorophyll fluorescence as a sensitive, non-destructive indicator to salinity [47, 48].

Different acid modification treatments likely induced distinct physicochemical changes in biochar, leading to varying biological effects [49]. Strong inorganic acids such as sulfuric acid may remove ash content and expose more active surface sites, thereby significantly enhancing ion exchange capacity and adsorption performance [50]. In contrast, organic acids such as citric acid may introduce functional groups in a milder manner, resulting in moderate improvements [51]. These differences provide a plausible explanation for the superior performance of BS compared with other treatments, highlighting the importance of modification strategy in determining biochar functionality.

Changes in pigment concentration further support these conclusions. The BS significantly increased chlorophyll and carotenoid contents. Carotenoids, key photoprotective pigments, help dissipate excess excitation energy and reduce the risk of photoinhibition [52]. The coordinated rise in chlorophylls and carotenoids improved light harvesting and energy distribution, enabling sugar beet seedlings to sustain higher photosynthetic activity under saline–alkali conditions [53, 54]. The larger leaf area observed under BS treatment also supports this interpretation, indicating that sulfuric acid–modified biochar enhances metabolic efficiency and promotes greater nutrient allocation to leaf development.

Saline–alkali stress often induces ROS overaccumulation, which impairs photosynthetic machinery and accelerates membrane damage [55–57]. In our study, unmodified biochar (B) produced no significant changes in SOD, POD, or CAT activities relative to CK, suggesting limited ROS regulation. In contrast, BS significantly reduced SOD activity without significantly affecting POD activity, which suggests that BS may potentially alleviate ROS production at an early stage, rather than relying on elevated enzymatic scavenging as the primary strategy for ROS detoxification. This response may result from increased surface functional groups and greater cation-exchange capacity in the modified biochar, which improves rhizosphere ion balance and reducing the ROS bursts triggered by ionic toxicity [58, 59].

Relative electrical conductivity and MDA content are important indicators of cell membrane permeability and lipid peroxidation [60, 61]. The BS treatment effectively lowered both parameters, suggesting enhanced membrane stability. This protection likely arises from improved ion homeostasis and osmotic adjustment rather than directly reducing lipid peroxidation [62, 63]. In addition, BS slightly increased soluble sugar content, an important osmolyte that contributes to osmotic balance, membrane stabilization, and protein protection [64]. Another potential mechanism is that sulfuric acid-modified biochar can enhance soil water retention and cation exchange capacity, which may help establish a more buffered rhizosphere environment. These improved conditions could assist in preserving stomatal function and reducing stomatal limitations under saline-alkali stress, thereby facilitating a more stable CO_2_ supply for photosynthesis [65].

In saline–alkali conditions, the whole-plant adaptation is dependent on the coordinated regulation of photosynthesis and antioxidant defense pathways [66–68]. The PLS-PM results in this study provided structural evidence supporting this plausible integrated mechanism. Sulfuric acid–modified biochar not only directly enhanced photosynthetic efficiency and chlorophyll fluorescence traits via elevated pigment accumulation, but also favouring overall plant growth. These findings are in line with previous studies indicating that chlorophyll content, one of the key indices influencing photosynthetic capacity under saline–alkali stress, contributes to biomass accumulation by modulating light harvesting and electron transport processes [69, 70]. Osmotic adjustment compounds also play a central part in regulating cellular homeostasis under stress. Their synergistic relationship with photosynthetic pigments, as indicated by the model, suggests that modified biochar enhances both photochemical processes and osmotic regulation, thereby strengthening whole-plant adaptability [71].

Nevertheless, this study has several limitations due to technical and time constraints. The physicochemical properties of the modified biochars were not directly characterized, which limits the ability to establish a detailed and mechanistic linkage between material properties and biological responses. Furthermore, the experiment was conducted as a short-term pot study at the seedling stage, which may not fully represent field conditions or later growth stages. Future studies should integrate detailed biochar characterization, rhizosphere soil analysis, and plant ionomic profiling to establish a comprehensive mechanistic framework. Long-term experiments, including field trials and multi-stage assessments, are needed to evaluate the stability and scalability of acid-modified biochar applications in saline–alkali soils.

## Conclusions

This study demonstrated that under saline–alkali conditions, the 2% sulfuric acid–modified biochar (BS) restored sugar beet seedling growth and sustained high photosynthetic performance. BS enhanced the levels of foliar chlorophyll and carotenoid contents, thereby strengthening light harvesting and overall photosynthetic efficiency. The improved photosynthesis increased plant height, leaf area, and aboveground biomass. Specifically, chlorophyll fluorescence measurements showed higher photosynthetic performance index (PI_ABS_) and maximum photochemical efficiency (F_v_/F_m_). The absence of distinct L- and K-bands in the OJIP curves further indicated that the foliar PSII structural integrity and electron transport were well maintained. In addition, BS reduced leaf relative EC and ROS accumulation, suggesting improved membrane integrity and reduced oxidative damage. Overall, the sulfuric acid–modified biochar alleviated the negative effects of saline–alkali stress by enhancing PSII functionality, improving light-energy allocation, and stabilizing cellular membranes, thereby restoring the physiology of sugar beet plants growing in otherwise unfavourable soils.

## Supplementary Information

Supplementary Material 1.

## Data Availability

Data are available from the corresponding authors on reasonable request. Prof Jean Yong (jean.yong@slu.se) and Prof Baiquan Song (13212929229@163.com).

## Abbreviations

BS: Sulfuric acid-modified biochar
BN: Nitric acid-modified biochar
BP: Phosphoric acid-modified biochar
BCA: Citric acid-modified biochar
BFA: Fulvic acid-modified biochar
PLS-PM: Partial least squares path modeling
POD: Peroxidase
SOD: Superoxide dismutase
CAT: Catalase
Pn: Net photosynthetic rate
Ci: Intercellular CO_2_ concentration
Gs: Stomatal conductance
E: Transpiration rate

## References

[1] Anonymous. Report from the Commission on the agreements within the trade for the sugar sector. In: European Commission Agriculture and rural development report. 2025. https://agriculture.ec.europa.eu/farming/crops/sugar_en.

[2] Anonymous. Agricultural OECD-FAO. Outlook 2024–2033. In: OECD-FAO Agricultural Outlook 2024–2033; Rome: Edited by Paris/FAO; Paris/FAO. 2024. https://openknowledge.fao.org/items/91c62ae8-b070-4ea8-8281-592376520b03.

[3] Munns R, Tester M. Mechanisms of Salinity Tolerance. Annu Rev Plant Biol. 2008;59(8):651–81. 18444910 10.1146/annurev.arplant.59.032607.092911

[4] Chen X, Yang S, Wen X. Simulation of sugar beet growth in saline-alkali land: Controlling the synergistic effect of aluminum sulfate irrigation on photosynthesis. Eur J Agron. 2025;172(87):127851.

[5] Yamada M, Yamada S, Fujiwara N, Kaburagi E, Murillo-Amador B. Effect of sodium on iron absorption, translocation and allocation in halophilic Amaranthaceae species. Plant Physiol Biochem. 2025;229(5):110597. 41398751 10.1016/j.plaphy.2025.110597

[6] Wang G, Ni G, Feng G, Burrill HM, Li J, Zhang J, Zhang F. Saline-alkali soil reclamation and utilization in China: progress and prospects. Front Agricultural Sci Eng. 2024;11(2):216–28.

[7] Wang Z, Yang Z, Liu L, Ye Y, Xie X. Electron transfer mechanism of chitosan-modified natural manganese ore-cornstalk biochar composites with activated peroxymonosulfate: The role of functional groups on the surface of biochar-based composites. Sep Purif Technol. 2022;302(77):11–3.

[8] Wang Y, Lin Q, Liu Z, Liu K, Wang X, Shang J. Salt-affected marginal lands: a solution for biochar production. Biochar. 2023;5(1):3–4.

[9] Blanco-Canqui H. Does biochar application alleviate soil compaction? Review and data synthesis. Geoderma. 2021;404(8):115317.

[10] Enebe MC, Ray RL, Griffin RW. Carbon sequestration and soil responses to soil amendments – a review. J Hazard Mater Adv. 2025;18(57):100714.

[11] Lepore E, Bondi G, Fenton O, Schmidt O, Tracy S, Wall DP. The power of soil amendments to restore degraded grassland soil: a combined approach using physical indicators and X-ray computed tomography. Soil Tillage Res. 2025;253(5):106674.

[12] Zhao W, Xiao J, Wang S, Gai X, Chen G. Bone biochar and humic acid improved soil quality and promoted *Olea europaea* growth in coastal saline soil by enhancing the stoichiometric homeostasis of nutrient elements. Biochar. 2025;7(1):88–90.

[13] Yildiztugay E, Ozfidan-Konakci C, Arikan B, Alp-Turgut FN, Gulenturk C. The regulatory effects of biochar on PSII photochemistry, antioxidant system and nitrogen assimilation in Lemna minor exposed to inorganic pollutants, arsenic and fluoride. J Environ Chem Eng. 2023;11(5):45127.

[14] Acharya BS, Dodla S, Wang JJ, Pavuluri K, Darapuneni M, Dattamudi S, Maharjan B, Kharel G. Biochar impacts on soil water dynamics: knowns, unknowns, and research directions. Biochar. 2024;6(1):68.

[15] Wang Z, Yang Z, Liu L, Ye Y, Xie X. Electron transfer mechanism of chitosan-modified natural manganese ore-cornstalk biochar composites with activated peroxymonosulfate: The role of functional groups on the surface of biochar-based composites. Sep Purif Technol. 2022;302(22):33–9.

[16] Li S, Harris S, Anandhi A, Chen G. Predicting biochar properties and functions based on feedstock and pyrolysis temperature: a review and data syntheses. J Clean Prod. 2019;215(5):890–902.

[17] Tomczyk A, Sokołowska Z, Boguta P. Biochar physicochemical properties: pyrolysis temperature and feedstock kind effects. Reviews Environ Sci Bio/Technology. 2020;19(1):191–215.

[18] Guan R, Li Y, Jia Y, Jiang F, Li L. Acidified biochar one-off application for saline-alkali soil improvement: a three-year field trial evaluating the persistence of effects. Ind Crops Prod. 2024;222:119972.

[19] Liu Z, Li Z, Chen S, Zhou W. Enhanced phytoremediation of petroleum-contaminated soil by biochar and urea. J Hazard Mater. 2023;453(5):131404. 37080026 10.1016/j.jhazmat.2023.131404

[20] Zhang G, Zhang L, Shi Z, Yang Y, Liu J. Microbial nutrient limitation and carbon use efficiency in saline-alkali soil amended with biochar: insights from ecoenzymatic C:N:P stoichiometry. Biochar. 2025;7(1):68.

[21] Wang S, Zhang T, Jahan I, Zhou Y, Li S, Cai Y, Cai K. Si-modified biochar mediated tomato resistance to bacterial wilt by increasing soil silicon availability and inducing plant defense. Plant Physiol Biochem. 2025;227(6):110163. 40543150 10.1016/j.plaphy.2025.110163

[22] Altaf MM, Ashraf S, Khan MQN, Sun X, Li D, El-Sheikh MA, Xu S, Zhu Z. Effectiveness of sulfur-modified wheat straw biochar in alleviating vanadium stress in rice: impacts on growth, photosynthesis, and redox regulation. Biochar. 2025;7(1):7–9.

[23] Zhang Y, Kaiser E, Dutta S, Sharkey TD, Marcelis LFM, Li T. Short-term salt stress reduces photosynthetic oscillations under triose phosphate utilization limitation in tomato. J Exp Bot. 2024;75(10):2994–3008. 38436737 10.1093/jxb/erae089

[24] Xu Y, Wu S, Huang F, Huang H, Yi Z, Xue S. Biomodification of feedstock for quality-improved biochar: a green method to enhance the Cd sorption capacity of Miscanthus lutarioriparius-derived biochar. J Clean Prod. 2022;350(11):55–6.

[25] Ou W, Lan X, Guo J, Cai A, Liu P, Liu N, Liu Y, Lei Y. Preparation of iron/calcium-modified biochar for phosphate removal from industrial wastewater. J Clean Prod. 2022;383(61):19–29.

[26] Gong H, Zhao L, Rui X, Hu J, Zhu N. A review of pristine and modified biochar immobilizing typical heavy metals in soil: applications and challenges. J Hazard Mater. 2022;432(78):52134. 10.1016/j.jhazmat.2022.128668 35325861

[27] Ghorbani M, Konvalina P, Kopecký M, Kolář L. A meta-analysis on the impacts of different oxidation methods on the surface area properties of biochar. Land Degrad Dev. 2023;34(2):299–312.

[28] Wang X, Riaz M, Babar S, Eldesouki Z, Liu B, Xia H, Li Y, Wang J, Xia X, Jiang C. Alterations in the composition and metabolite profiles of the saline-alkali soil microbial community through biochar application. J Environ Manage. 2024;352(15):120033. 38218168 10.1016/j.jenvman.2024.120033

[29] Wang H, Guo Y, Fang H, Cheng S, Shi F, Chen L, Pu H, Liu B. Divergent effects of straw and straw-derived biochar on soil N transformation and N_2_O emissions: a global meta-analysis. J Environ Manage. 2025;395(5):66–9. 10.1016/j.jenvman.2025.127784 41166952

[30] Kumar Y, Ren W, Tao H, Tao B, Lindsey LE. Impact of biochar amendment on soil microbial biomass carbon enhancement under field experiments: a meta-analysis. Biochar. 2025;7(1):2. 10.1007/s42773-024-00391-6.

[31] Wright IJ, Reich PB, Westoby M, Ackerly DD, Baruch Z, Bongers F, Cavender-Bares J, Chapin T, Cornelissen JHC, Diemer M, et al. The worldwide leaf economics spectrum. Nature. 2004;428(6985):821–7. 15103368 10.1038/nature02403

[32] Wu Z, Wang X, Song B, Zhao X, Du J, Huang W. Responses of photosynthetic performance of sugar beet varieties to foliar boron spraying. Sugar Tech. 2021;23(6):1332–9.

[33] Linn AI, Zeller AK, Pfündel EE, Gerhards R. Features and applications of a field imaging chlorophyll fluorometer to measure stress in agricultural plants. Precision Agric. 2021;22(3):947–63.

[34] Beauchamp C, Fridovich I. Superoxide dismutase: Improved assays and an assay applicable to acrylamide gels. Anal Biochem. 1971;44(1):276–87. 4943714 10.1016/0003-2697(71)90370-8

[35] Cai K, Gao D, Luo S, Zeng R, Yang J, Zhu X. Physiological and cytological mechanisms of silicon-induced resistance in rice against blast disease. Physiol Plant. 2008;134(2):324–33. 18513376 10.1111/j.1399-3054.2008.01140.x

[36] Aebi H. Catalase. In: Methods of Enzymatic Analysis (Second Edition). Edited by Bergmeyer HU: Academic Press. 1974;2:673–684.

[37] De Vos CHR, Schat H, De Waal Ma, Vooijs M. Ernst W H O: Increased resistance to copper-induced damage of the root cell plasmalemma in copper tolerant Silene cucubalus. Physiol Plant. 1991;82(4):523–8.

[38] Ebell LF. Variation in total soluble sugars of conifer tissues with method of analysis. Phytochemistry. 1969;8(1):227–33.

[39] Baker NR. Chlorophyll fluorescence: a probe of photosynthesis in vivo. Annu Rev Plant Biol. 2008;59(1):89–113. 18444897 10.1146/annurev.arplant.59.032607.092759

[40] Rasouli F, Kiani-Pouya A, Tahir A, Shabala L, Chen Z, Shabala S. A comparative analysis of stomatal traits and photosynthetic responses in closely related halophytic and glycophytic species under saline conditions. Environ Exp Bot. 2021;181(77):104300.

[41] Sultan H, Mazhar Abbas HM, Faizan M, Emamverdian A, Shah A, Bahadur S, Li Y, Khan MN, Nie L. Residual effects of biochar and nano-modified biochar on growth and physiology under saline environment in two different genotype of *Oryza sativa* L. J Environ Manage. 2025;373(5):66–9. 10.1016/j.jenvman.2024.123847 39746259

[42] Lewis CM, Flory JD, Moore TA, Moore AL, Rittmann BE, Vermaas WFJ, Torres CI, Fromme P. Electrochemically driven photosynthetic electron transport in cyanobacteria lacking photosystem II. J Am Chem Soc. 2022;144(7):2933–42. 35157427 10.1021/jacs.1c09291

[43] Schonknecht G, Neimanis S, Katona E, Gerst U, Heber U. Relationship between photosynthetic electron transport and pH gradient across the thylakoid membrane in intact leaves. Proc Natl Acad Sci USA. 1995;55(9):12–28. 10.1073/pnas.92.26.12185 PMC40321 11607620

[44] Slatni T, Ben Slimene I, Harzalli Z, Taamalli W, Smaoui A, Abdelly C, Elkahoui S. Enhancing quinoa (*Chenopodium quinoa*) growth in saline environments through salt-tolerant rhizobacteria from halophyte biotope. Physiol Plant. 2024;176(4):135456. 10.1111/ppl.14466 39164839

[45] Chen S, Li Y, Zhang W, Shen H, Wang S, Wang R, Li H. Evaluation of saline-alkali tolerance of *Oenothera *L. germplasms and their morpho‐physiological responses to saline‐alkali stress. Physiol Plant. 2025;177(5):32591. 10.1111/ppl.70541 41035118

[46] Kumari A, Fatnani D, Seth CS, Parida AK. Unravelling the metabolic signatures and associated pathways underlying saline-alkali stress resilience in the halophyte *Salvadora persica*. Physiol Plant. 2025;177(1):e70114. 10.1111/ppl.70114. 10.1111/ppl.70114 39962363

[47] Kumari A, Fatnani D, Seth CS, Parida AK. Unravelling the metabolic signatures and associated pathways underlying saline-alkali stress resilience in the halophyte *Salvadora persica*. Physiol Plant. 2025;177(1):19–24. 10.1111/ppl.70114 39962363

[48] Thomas SC, Frye S, Gale N, Garmon M, Launchbury R, Machado N, Melamed S, Murray J, Petroff A, Winsborough C. Biochar mitigates negative effects of salt additions on two herbaceous plant species. J Environ Manage. 2013;129(5):62–8. 23796889 10.1016/j.jenvman.2013.05.057

[49] Sierra-Jimenez V, Macias RJ, Mathews JP, Carré V, Leclerc S, Budai A, Chejne F, Castro-Gutiérrez J, Celzard A, Fierro V, et al. Influence of acid-catalyzed dehydration and pressure on woody biomass carbonization: Exploring carbon yield, heteroatom functionalities, and biochar atomistic structure. Carbon. 2025;242(7):120474.

[50] Peiris C, Nayanathara O, Navarathna CM, Jayawardhana Y, Nawalage S, Burk G, Karunanayake AG, Madduri SB, Vithanage M, Kaumal MN, et al. The influence of three acid modifications on the physicochemical characteristics of tea-waste biochar pyrolyzed at different temperatures: a comparative study†. RSC Adv. 2019;9(31):17612–22. 35520596 10.1039/c9ra02729g PMC9064594

[51] Jiao M, Shi Y, Li M, Zhang H, Li S, Deng H, Xia D. The surface functional groups-driven fast and catalytic degradation of naproxen on sludge biochar enhanced by citric acid. Environ Pollut. 2024;361(10):124857. 39214447 10.1016/j.envpol.2024.124857

[52] Demmig-Adams B, Adams WW. The role of xanthophyll cycle carotenoids in the protection of photosynthesis. Trends Plant Sci. 1996;1(1):21–6.

[53] Fink J, Sánchez-Rodríguez AR, Frosi G, Eckert D, Bonetti JA, Bastiani K, Lavratti A, Inda AV, Zanquetti A. Industrial saline wastewater in a corn-soybean rotation to enhance crop yield without compromising soil health in a subtropical soil. J Environ Manage. 2021;296(72):98–107. 10.1016/j.jenvman.2021.113341 34351294

[54] Zarea MJ, Hajinia S, Karimi N, Mohammadi Goltapeh E, Rejali F, Varma A. Effect of *Piriformospora indica* and *Azospirillum* strains from saline or non-saline soil on mitigation of the effects of NaCl. Soil Biol Biochem. 2011;45(3):139–46.

[55] Dawood MFA, Sohag AAM, Tahjib-Ul-Arif M, Abdel Latef. A A H: Hydrogen sulfide priming can enhance the tolerance of artichoke seedlings to individual and combined saline-alkaline and aniline stresses. Plant Physiol Biochem. 2021;159(37):347–62. 33434783 10.1016/j.plaphy.2020.12.034

[56] Wang Y, Wang J, Guo D, Zhang H, Che Y, Li Y, Tian B, Wang Z, Sun G, Zhang H. Physiological and comparative transcriptome analysis of leaf response and physiological adaption to saline alkali stress across pH values in alfalfa (*Medicago sativa*). Plant Physiol Biochem. 2021;167(8):140–52. 34352517 10.1016/j.plaphy.2021.07.040

[57] Wang Z, Li Z, Wang Z, Liu T, Zhang P, Li S, Ye S, Yang K, Gai Z, Liu L. Alkaline stress suppresses soybean waterlogging tolerance by exacerbating energy expenditure and ROS accumulation. Plant Physiol Biochem. 2025;229:110381. 40829340 10.1016/j.plaphy.2025.110381

[58] Ji X, Lin H, Zhang Y, Lu J, Zhang Y, Sun H, Wang G, Yang X, Li W, Duan J, et al. Activation of peroxymonosulfate by magnesium–modified biochar for the degradation of imidacloprid: Effectiveness and mechanisms. Chem Eng J. 2025;524(39):55–9.

[59] Khan ZH, Yu H, Li H, Hassan Kazmi SS, U, Han R, Azeem M, Sun X, Rehman ZU, Li G. Fe-modified biochar-driven ROS generation in the rhizosphere and their role in microplastic transformation. J Hazard Mater. 2025;497:139718. 10.1016/j.jhazmat.2025.139718. 10.1016/j.jhazmat.2025.139718 40907322

[60] Lin Y, Chen G, Lin H, Lin M, Wang H, Lin Y. Chitosan postharvest treatment suppresses the pulp breakdown development of longan fruit through regulating ROS metabolism. Int J Biol Macromol. 2020;165(9):601–8. 33002534 10.1016/j.ijbiomac.2020.09.194

[61] Shen X, Liu Y, Zeng Y, Zhao Y, Bao Y, Shao X, Wu Z, Zheng Y, Jin P. Hydrogen sulfide attenuates chilling injury in loquat fruit by alleviating oxidative stress and maintaining cell membrane integrity. Food Chem. 2024;463(9):95481. 10.1016/j.foodchem.2024.141094 39270496

[62] Farhangi-Abriz S, Torabian S. Antioxidant enzyme and osmotic adjustment changes in bean seedlings as affected by biochar under salt stress. Ecotoxicol Environ Saf. 2016;137(37):64–70. 27915144 10.1016/j.ecoenv.2016.11.029

[63] Ju J, Xie Y, Yu H, Guo Y, Cheng Y, Zhang R, Yao W. Synergistic inhibition effect of citral and eugenol against *Aspergillus niger *and their application in bread preservation. Food Chem. 2019;310(5):78–9. 10.1016/j.foodchem.2019.125974 31835216

[64] Yang T, Gan L, Peng X, Peng X, Li L, Huang Y, Xiong F, Wei M. Overexpression of cassava MeAMY1 and MeBAM3 genes enhance drought and salt stress tolerance in transgenic *Arabidopsis*. Plant Physiol Biochem. 2025;226(5):4247. 10.1016/j.plaphy.2025.110058 40413961

[65] Song X, Li H, Song J, Chen W, Shi L. Biochar/vermicompost promotes hybrid *Pennisetum* plant growth and soil enzyme activity in saline soils. Plant Physiol Biochem. 2022;183(9):96–110. 35576892 10.1016/j.plaphy.2022.05.008

[66] Feng N, Yu M, Li Y, Jin D, Zheng D. Prohexadione-calcium alleviates saline-alkali stress in soybean seedlings by improving the photosynthesis and up-regulating antioxidant defense. Ecotoxicol Environ Saf. 2021;220(84):112369. 34090109 10.1016/j.ecoenv.2021.112369

[67] Lu W, Wei G, Zhou B, Liu J, Zhang S, Guo J. A comparative analysis of photosynthetic function and reactive oxygen species metabolism responses in two hibiscus cultivars under saline conditions. Plant Physiol Biochem. 2022;184(8):87–97. 35636335 10.1016/j.plaphy.2022.05.023

[68] Jakada BH, Zhu F, Waqas K, Chen H, Guan Q, Lan X. The CBL-interacting protein kinase 9 (OsCIPK9) contributes to saline-alkali stress tolerance in rice. Plant Physiol Biochem. 2025;229(79):99–101. 10.1016/j.plaphy.2025.110332 40763554

[69] Zhang Y, Kaiser E, Marcelis LFM, Yang Q, Li T. Salt stress and fluctuating light have separate effects on photosynthetic acclimation, but interactively affect biomass. Plant Cell Environ. 2020;43(9):2192–206. 32463133 10.1111/pce.13810

[70] ElSayed AI, Rafudeen MS, Gomaa AM, Hasanuzzaman M. Exogenous melatonin enhances the reactive oxygen species metabolism, antioxidant defense-related gene expression, and photosynthetic capacity of *Phaseolus vulgaris* L. to confer salt stress tolerance. Physiol Plant. 2021;173(4):1369–81. 33619766 10.1111/ppl.13372

[71] Rawat N, Wungrampha S, Singla-Pareek SL, Yu M, Shabala S, Pareek A. Rewilding staple crops for the lost halophytism: Toward sustainability and profitability of agricultural production systems. Mol Plant. 2021;15(1):45–64. 34915209 10.1016/j.molp.2021.12.003

