# Supplementary material for: Acid-modified biochar improves the adaptability of sugar beet seedlings to saline-alkali soil by enhancing their physiology
Source: BMC Plant Biol. 2026 Aug 20;26:1425. doi: 10.1186/s12870-026-09769-z (PMC13491937; doi:10.1186/s12870-026-09769-z)
Supplement: Supplementary file 1 [file 12870_2026_9769_MOESM1_ESM.docx]

**Supplementary materials**

**Contents of Supplementary Material**

**Table S1** Soil physicochemical properties

**Table S1** Soil physical and chemical properties for growing sugar beet

| Treatment | CK | B | BS | BCA |
| --- | --- | --- | --- | --- |
| pH | 8.09±0.18a | 8.00 ±0.19a | 7.75 ±0.11b | 7.83 ±0.29ab |
| EC  (ds m^-1^) | 5.34±0.21a | 5.38±0.15a | 4.69±0.76b | 5.98±0.43a |
| AN  (mg kg^-1^) | 171.50±10.52c | 199.05±10.10c | 264.86±16.65b | 328.83±19.74a |
| AP  (mg kg^-1^) | 40.05±0.27a | 46.59±1.29a | 46.33±8.49a | 44.11±4.01a |
| AK  (mg kg^-1^) | 256.41±16.16a | 252.21±27.09a | 255.19±11.99a | 244.81±19.08a |

Note: Data are expressed as the mean +/- standard deviation (SD) of three biological replicates per treatment. The change in lowercase letters indicates statistically significant differences (*P* <0.05).
